# Measuring endogenous changes in serotonergic neurotransmission with [^11^C]Cimbi-36 positron emission tomography in humans

**DOI:** 10.1038/s41398-019-0468-8

**Published:** 2019-04-11

**Authors:** Sofi da Cunha-Bang, Anders Ettrup, Brenda Mc Mahon, Anine Persson Skibsted, Martin Schain, Szabolcs Lehel, Agnete Dyssegaard, Louise Møller Jørgensen, Kirsten Møller, Nic Gillings, Claus Svarer, Gitte M. Knudsen

**Affiliations:** 1 Neurobiology Research Unit and Center for Integrated Molecular Brain Imaging (Cimbi), Copenhagen University Hospital, Rigshospitalet, Blegdamsvej 9, Copenhagen, 2100 Denmark; 2 PET and Cyclotron Unit, Copenhagen University Hospital, Rigshospitalet, Blegdamsvej 9, Copenhagen, 2100 Denmark; 3 Department of Neuroanaesthesiology, Copenhagen University Hospital, Rigshospitalet, Blegdamsvej 9, Copenhagen, 2100 Denmark

## Abstract

Developing positron emission tomography (PET) radioligands for the detection of endogenous serotonin release will enable the investigation of serotonergic deficits in many neuropsychiatric disorders. The present study investigates how acute challenges that aim to increase or decrease cerebral serotonin levels affect binding of the serotonin 2A receptor (5-HT_2A_R) agonist radioligand [^11^C]Cimbi-36. In a randomized, double-blind, placebo-controlled, three-arm design, 23 healthy volunteers were PET scanned twice with [^11^C]Cimbi-36: at baseline and following double-blind assignment to one of three interventions (1) infusion of the selective serotonin reuptake inhibitor (SSRI) citalopram preceded by oral dosing of the 5-HT_1A_R antagonist pindolol, (*n* = 8) (2) acute tryptophan depletion (ATD) (*n* = 7) and (3) placebo (*n* = 8). Two-sample t-tests revealed no significant group differences in percent change of neocortical [^11^C]Cimbi-36 binding from baseline to intervention between placebo and citalopram/pindolol (*p* = 0.4) or between placebo and ATD (*p* = 0.5). Notably, there was a significantly larger within-group variation in 5-HT_2A_R binding after intervention with citalopram/pindolol, as compared with placebo (*p* = 0.007). These findings suggest that neither ATD nor a combination of citalopram and pindolol elicit acute unidirectional changes in serotonin levels sufficient to be detected with [^11^C]Cimbi-36 PET in neocortex. We suggest that the large interindividual variation in 5-HT_2A_R binding after citalopram/pindolol reflects that after an acute SSRI intervention, individuals respond substantially different in terms of their brain serotonin levels. Our observation has a potential impact for the understanding of patient responses to SSRI.

## Introduction

Serotonergic neurotransmission is implicated in cognitive and emotional processes, and altered serotonin signalling is thought to contribute to a variety of disorders such as depression, addiction, schizophrenia, and anxiety. The development of novel methods to examine the serotonin system in the human brain is important because they can give important insights into the serotonergic mechanisms involved in the pathophysiology of neuropsychiatric disorders.

Positron emission tomography (PET) is a neuroimaging technique with unsurpassed selectivity and sensitivity to quantify neuroreceptors in vivo. PET can be used to measure dynamic changes in neurotransmission that occur in response to endogenous fluctuations in neurotransmitter levels or to experimental challenges^1^. Appropriate PET radioligands enables the detection of endogenous neurotransmitter release, i.e. radioligand binding is inversely correlated with in vivo concentration of neurotransmitters^2^. Radioligand agonists are hypothesised to be superior to antagonists for the detection of acute neurotransmitter release because of differential receptor-affinity states^2^. For the dopamine system, it has been reported that D2/D3 receptor agonist radioligands are more sensitive to endogenous changes in dopamine than antagonist radioligands^3^. This difference has been explained by agonist radioligands binding to the high affinity state receptor only, inducing competition with endogenous neurotransmitters^2^. Accordingly, it was recently shown in non-human primates that the serotonin 2A (5-HT_2A_) receptor agonist radioligand [^11^C[Cimbi-36 is more sensitive to serotonin release than the antagonist 5-HT_2A_ receptor radioligand [^11^C]MDL 100907^4^.

In humans, several PET serotonergic radioligand studies have failed to show convincing radioligand displacement after drug-induced changes in brain serotonin levels^2^. Two studies with the partial agonist radiotracer for the 5-HT_1A_ receptor ([^11^C]CUMI) found either no change^5^ or an unexpected increase in radioligand binding^6^. Likewise, the binding of the 5-HT_1B_ partial agonist radioligand [^11^C]AZ10419369 increased after an acute challenge with a selective serotonin reuptake inhibitor (SSRI) in humans^7^. It was suggested that the unexpected *increase* in radiotracer binding in postsynaptic regions was caused by stimulation of the 5-HT_1A_ and 5-HT_1B_ autoreceptors in the raphe nuclei, which led to a subsequent inhibition of serotonin release in the terminals^6,7^.

The aim of this study was to assess the sensitivity of [^11^C]Cimbi-36 binding to acute changes in serotonin levels in the healthy human brain. In a pseudo-randomized, double-blind, placebo-controlled, 3-arm design, healthy volunteers were scanned with [^11^C]Cimbi-36 PET at baseline and following intervention with either placebo, the SSRI citalopram combined with a 5-HT_1A_ receptor antagonist pindolol, or acute tryptophan depletion (ATD). We hypothesised that citalopram/pindolol would increase brain serotonin levels and be associated with a decrease in [^11^C]Cimbi-36 binding and conversely, that ATD would decrease brain serotonin levels and thereby increase [^11^C]Cimbi-36 binding.

## Materials and methods

### Study design

This randomized double-blind, placebo-controlled intervention study was approved by the Ethics committee for the Capital Region of Denmark (Journal number: H-4-2012-105), and the first-in-human use of [^11^C]Cimbi-36 was approved by the Danish Health and Medicines Authority (EudraCT number: 2012-002056-16). Prior to initiation, the trial was registered for public access at clinicaltrials.gov (Identifier: NCT01778686). Twenty-four healthy volunteers were PET scanned twice with [^11^C]Cimbi-36 at baseline and following random assignment to one of three groups: eight subjects received the SSRI citalopram in combination with the 5-HT_1A_ receptor antagonist pindolol, eight subjects received ATD and eight subjects received placebo. Sample size was not based on test-retest [^11^C]Cimbi-36, because this study was part of the first study done in humans with [^11^C]Cimbi-36. The study took place at the Neurobiology Research Unit (NRU), Department of Radiology and PET and Cyclotron Unit of the Copenhagen University Hospital Rigshospitalet, Copenhagen, Denmark, from January to October 2013.

### Subjects

Healthy volunteers were recruited through online advertisements (www.forsogsperson.dk) and paper advertisements on bulletin boards at universities. Interested individuals signed up electronically informing their contact details, age, gender and demographic variables, which are stored in a database accessible for researchers at NRU to enroll possible participants for brain scanning studies. Exclusion criteria were current or lifetime history of psychiatric disorder, medical or neurological disease, severe head trauma, alcohol or substance abuse, as well as severe visual or hearing impairment and contraindications for MRI. No participant had any medical or neurological illness according to history, routine blood biochemistry and physical examination, evaluated on the day of scanning. The participants took no medications, except for seven women who used hormonal contraceptives and one participant who reported taking antihistamines. None of the participants had been exposed to psychotropic medications. All had a negative on urine drug screen (Rapid Response Multi-Drug; BTNX Inc., Toronto, Ontario, Canada), and all female subjects had a negative urine pregnancy test on the day of scanning. The participants showed no signs of psychopathology according to the Symptom Checklist Revised (SCL-92, Global Severity Index score, mean score ± SD: 0.21 ± 0.21)^8^. All participants provided written informed consent following full description of the procedures and received monetary compensation for their participation. One subject in the ATD group was excluded from the analysis after suffering an allergic reaction during the intervention scan. This subject was referred to an allergist consultant and examinations excluded that [^11^C]Cimbi-36 was the cause of the allergic reaction. Eight subjects (placebo group) were also included in a previous study evaluating the test-retest reliability of [^11^C]Cimbi-36^9^.

### Serotonergic interventions and procedure

Each subject participated in two PET experiments with [^11^C]Cimbi-36. The participants were scanned at baseline, and approximately four weeks later they were scanned following intervention or placebo. An overview and timing of the interventions are shown in Table 1. During PET scanning, subjects were monitored with 3-lead electrocardiography continuously and non-invasive measurements of oxygen saturation and blood pressure at regular intervals. In one of the PET scans (baseline scan, placebo group) the scanner failed to start correctly. The time course of the first three minutes of tissue time-activity curves from this scan were interpolated from the subject’s intervention scan using the shape of the uptake curve from the same subject intervention scan while scaling to the radioactive concentration obtained after the initial missing three minutes of the baseline scan.

**Table 1:** Timing of interventions, referenced to the time of [^11^C]Cimbi-36 rescan (injection time ranged from 12.29 to 13.05)

	−4 weeks	−3 days	−2 days	−1 day	−4.5 h	−4 h	−1.5 h	−30 min	0 min
Citalopram/Pindolol group	Baseline [^11^C]Cimbi-36 PET scan	Hexapindol 2.5 mg × 3	Hexapindol 5 mg × 3	Hexapindol 7.5 mg × 3	Hexapindol 7.5 mg × 1	AA drink with Trp	Hexapindol 7.5 mg × 1	Iv infusion Seropram 40 mg	[^11^C]Cimbi-36 PET rescan
Placebo group	Baseline [^11^C]Cimbi-36 PET scan	Placebo tablets × 3	Placebo tablets × 3	Placebo tablets × 3	Placebo tablets × 1	AA drink with Trp	Placebo tablet x 1	Iv infusion saline	[^11^C]Cimbi-36 PET rescan
ATD group	Baseline [^11^C]Cimbi-36 PET scan	Placebo tablets × 3	Placebo tablets x 3	Placebo tablets × 3	Placebo tablets × 1	AA drink devoid of Trp	Placebo tablet × 1	Iv infusion saline	[^11^C]Cimbi-36 PET rescan

*AA* Amino acid, *Trp* tryptophan, *Iv* intravenous

**Table 2:** Demographics and [^11^C]Cimbi-36 radioligand injection parameters at baseline and intervention PET scans

	Placebo		Citalopram/Pindolol		ATD	
Number of subjects	8 (4 males)		8 (4 males)		8 (4 males)	
Age (years)	22.0 ± 2.5		21.9 ± 2.0		22.3 ± 3.2	
Body Mass Index (kg/m^2^)	23.6 ± 2.5		23.9 ± 1.9		22.6 ± 1.9	
Interscan interval (days)	30.6 ± 15.5		22.4 ± 2.8		27.6 ± 17.1	
	Baseline	Intervention	Baseline	Intervention	Baseline	Intervention^a^
Injected dose (MBq)	509 ± 95	581 ± 27	477 ± 142	574 ± 37	554 ± 54	554 ± 52
Injected mass (µg)	0.9 ± 0.5	0.9 ± 0.5	0.9 ± 0.6	0.7 ± 0.5	0.9 ± 0.5	1.0 ± 0.4
Free fraction in plasma, *f*_P_ (%)	2.7 ± 0.4	3.7 ± 0.5	2.9 ± 0.5	3.4 ± 0.8	3.0 ± 0.5	3.0 ± 0.8

Values represent mean ± standard deviation

^a^ One female subject excluded due to adverse reaction during the baseline scan

In order to elevate brain serotonin levels, eight subjects received an intravenous administration of citalopram immediately before the intervention scan (starting 30 minutes before the scan). Prior to this, these subjects received the 5-HT_1A/1B_ receptor antagonist pindolol (Hexapindol®) to block the autoreceptors and thus inhibit the raphe nuclei-mediated response that could decrease serotonergic firing rates in response to SSRI-induced rise in serotonin levels. The subjects were instructed by blinded investigators to take either placebo or pindolol in a dose escalation regime to minimize side effects. They took three daily pindolol doses of 2.5 mg, 5 mg, and 7.5 mg, three days, two days, and one day before the intervention day, respectively. On the intervention day, they took 7.5 mg pindolol in the morning and again approximately one hour before the [^11^C]Cimbi-36 scanning. The 16 subjects who were not randomized to this group received placebo tablets in a corresponding regime. The randomization and preparation of pindolol/placebo pre-treatment were undertaken by two unblinded research administrators, who were not otherwise involved in the study. Before the subjects left the research facilities after the baseline scan, they received an envelope containing Hexapindol® or placebo tablets along with written instructions of dosing, possible side effects and a contact number to a medical doctor in case of adverse reactions or intolerable side effects. To ensure compliance during this period, reminder text messages were sent to their mobile phones three times daily. Citalopram (Seropram® Lundbeck, Valby, Denmark) was administered intravenously as a constant infusion over one hour where subjects received 40 mg starting 30min before [^11^C]Cimbi-36 injection. The 16 subjects in the ATD and placebo groups received saline infusion. Infusions were prepared by an unblinded research nurse, but the infusions were blinded both to the participants and to the investigators.

To achieve a reduction in serotonin levels, eight subjects were randomized to ATD, which is a dietary method to lower plasma tryptophan and thereby reduce the amount available for central serotonin synthesis^10,11^. We used a previously published approach^10^, which involves ingestion of either a gelatin-based collagen peptide (CP) mixture (Solugel 5000®, PB Gelatins, Tessenderlo, Belgium) that is naturally lacking tryptophan (CP-Trp), or a CP mixture supplemented with tryptophan (CP+Trp) (Supplementary Figure S1 for full description of content). The CP-Trp drink was prepared by adding 200 ml water to 100 g Solugel 5000®. Fifty mL artificial flavoring and ice was also added to make the mixture palatable. The CP+Trp drink was prepared by adding 1.2 g of L-tryptophan (Sigma-Aldrich, Denmark) to the CP-Trp drink. The randomization and preparation of the CP mixtures were undertaken by unblinded research administrators who were not otherwise involved in the study. The subjects ingested the drink at the research facilities four hours prior to the intervention scan. The participants were instructed to avoid protein-rich food on the day before the intervention scan (Supplementary Methods), as well as to fast from 6 am on the intervention day. The 16 subjects in the citalopram/pindolol and placebo groups also followed the low protein diet, and were given a placebo drink four hours before the intervention scan (CP+Trp). Tryptophan passes the blood-brain-barrier by a specific carrier for which tryptophan competes with all other large neutral amino acids (LNAAs)^12^. All subjects received a standardized diet throughout the intervention day to ensure low tryptophan levels and low carbohydrate intake (Supplementary Methods), as insulin secretion preferentially stimulates uptake of all LNAAs except for tryptophan into tissue. This leads to depletion of LNAAs in plasma and consequently an increase in the ratio between tryptophan and the other LNAAs in plasma, which can result in elevated levels of tryptophan entering the brain^13^. That is, a high carbohydrate load can facilitate the entrance of tryptophan into the brain. During the intervention scan day, plasma levels of prolactin, citalopram, tryptophan and LNAAs were measured to verify the effects of the citalopram/pindolol and ATD interventions, respectively. Venous blood samples were analyzed for content of tryptophan and LNAAs using HPLC (Supplementary Methods). Tryptophan load was calculated as the ratio between tryptophan and the sum of the other LNAA (Trp/ΣLNAA ratio).

### Magnetic Resonance Imaging

All subjects underwent MRI scans in a 3T Siemens Magnetom Verio scanner (Siemens AG, Erlangen, Germany) using a Siemens 32-channel head coil. Structural T1- and T2-weighted images (T1 protocol: Isotropic 0.9 × 0.9 × 0.9 mm resolution, repetition time (TR) = 1900 ms, echo delay time (TE) = 2.32 ms, inversion time = 900 ms, and flip angle = 9 degrees. T2 protocol: Isotropic 1 × 1 × 1 mm resolution, TR = 3200 ms, TE = 409 ms) were recorded for each subject, and based on these, a segmented MR-image was produced with vbm8 in SPM8 (Welcome Department of Imaging Neuroscience, London, http://www.fil.ion.ucl.ac.uk/spm) to mask grey matter in the subsequent automated extraction of tissue time-activity curves.

### PET scanning

The 5-HT_2A_ receptor agonist PET radioligand [^11^C]Cimbi-36 was produced for human administration, as previously described^14^. All participants were scanned in a high resolution research tomography (HRRT) PET scanner (CTI/Siemens, Knoxville, TN). Following a ten minute transmission scan a two hour emission scan was started at the time of a [^11^C]Cimbi-36 bolus injection (mean injected activity: 500 ± 117 MBq; injected cold Cimbi-36 dose: 0.83 ± 0.51 µg). Attentuation correction was performed as previously described^15^. [^11^C]Cimbi-36 scanning data were reconstructed into 45 dynamic frames (6 × 10 s, 6 × 20 s, 6 × 60 s, 8 × 120 s, 19 × 300 s). All PET images were motion corrected using the AIR (Automated Image Registration, v. 5.2.5, LONI, UCLA, http://bishopw.loni.ucla.edu/air5/) software. All frames were aligned to the first 5-minute frame, and no partial-volume correction was applied. Tissue time-activity curves were automatically extracted from a set of 45 distinct regions of interest (ROIs) using a data pipeline similar to previously reported^16^. Briefly, unfiltered PET images were coregistered and aligned to the subjects’ T1-weighted MRI image in SPM. Coregistration and ROI placement were visually inspected for each subject by overlaying ROIs on PET and MRI images. A global neocortical region was defined as volume-weighted means of the cortical ROIs: orbitofrontal cortex, superior, middle and inferior frontal gyrus; superior, middle and inferior temporal gyrus, sensorimotor cortex, parietal cortex and occipital cortex. The striatum was defined as volume-weighted means of putamen and caudate.

Radiometabolite analyses were performed using high-performance liquid chromatography (HPLC) in order estimate the concentrations of parent compound in arterial plasma, which were used as input functions in subsequent kinetic modeling. During the first 10 minutes after injection, radioactivity in whole blood was continuously measured in 2-second intervals using an Allogg ABSS autosampler (Allogg Technology, Mariefred, Sweden) counting coincidences in a lead-shielded detector (flow: 8 mL/min). Also, blood samples were drawn manually 2.5, 5, 10, 15, 20, 30, 40, 50, 70, 90, 105, and 120 minutes after injection. Plasma was obtained by centrifugation (1,500 × *g* for 7 minutes at 4 °C) of arterial whole blood. Radioactivity in whole blood and plasma was measured using a well counter (Cobra 5003; Packard Instruments, Meriden, CT, USA), which was cross-calibrated to the high-resolution research tomography scanner and to the autosampler. All samples were decay corrected to the time of radioligand injection.

### Quantification of [^11^C]Cimbi-36 binding

Kinetic modeling was performed in PMOD version 3.0 (PMOD Technologies Inc.). The investigators were blinded to which intervention group the participants were allocated to, until data analysis was complete. The regional binding potential (BP_ND_) of [^11^C]Cimbi-36 was calculated using the simplified reference tissue model (SRTM)^14^, and used as the primary outcome measure for the main statistical analyses. Secondary outcome measures included regional BP_ND_s that were calculated from the total distribution volumes (V_T_) derived from arterial input measurements and 2-tissue compartment modeling (2-TCM) as (V_T_^target^−V_T_^cerebellum^)/V_T_^cerebellum^. Results using 2-TCM are presented in supplemental information. We did not find BP_P_ suitable as an outcome measure due to changes in free radioligand fraction in plasma (*f*_P_) as described in the results section and Supplementary Figure S8.

### Statistical analyses

From [^11^C]Cimbi-36 BP_ND_s, the percentage difference between baseline and intervention scans was calculated as (BP_ND_^intervention^ – BP_ND_^baseline^)/BP_ND_^baseline^ for each subject. We used neocortex as our primary region of interest based on our hypothesis that the interventions would have global effects on serotonin levels. Also, neocortex is a high binding region of 5-HT_2A_ receptors. Differences in percent change of neocortical [^11^C]Cimbi-36 BP_ND_ between groups (citalopram/pindolol vs. placebo, and ATD vs. placebo) were assessed with unpaired *t*-tests (two-tailed, unequal variances). Normality of the data evaluated in main analyses was assessed with Shapiro-Wilk normality tests, which did not indicate substantial violation of assumptions (all *p* > 0.2). Homogeneity of variance was evaluated using Levene’s test. Post-hoc we analyzed group differences in cortical and hippocampal regional [^11^C]Cimbi-36 BP_ND_. We also evaluated at thalamus and striatum but did not find these regions suitable for evaluating group differences because we observed a spurious increase in BP_ND_ from baseline to intervention in the placebo group (of 20% and 15%, respectively^9^).

The effects of the interventions on prolactin and tryptophan concentrations were evaluated using a between-group two-way analysis of variance (ANOVA) of concentrations with time and treatment as main effects. Differences between groups at each timepoint were assessed with Bonferroni-corrected t-tests. The mean absolute difference in neocortical [^11^C]Cimbi-36 BP_ND_ between baseline and placebo intervention was previously reported to be 9% and 4% with 2-TCM and SRTM, respectively^9^.

## Results

**Fig. 1:**
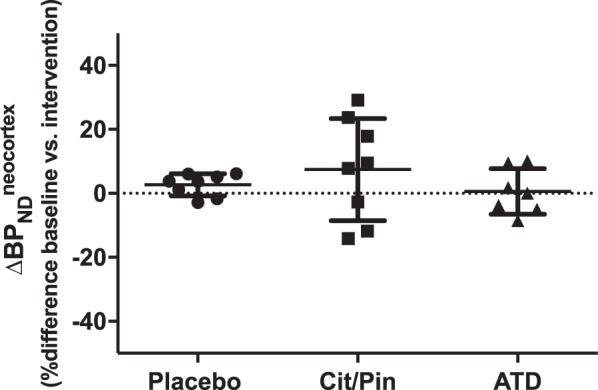
Effects of citalopram/pindolol (Cit/Pin) and acute tryptophan depletion (ATD) intervention on [^11^C]Cimbi-36 binding potential (BP_ND_) in neocortex. Difference in outcome is calculated as (BP_ND_^intervention^−BP_ND_^baseline^)/ BP_ND_^baseline^ for each subject

Demographics and [^11^C]Cimbi-36 radioligand injection parameters are presented in Table 2. The mean percent differences (±SD) in neocortical [^11^C]Cimbi-36 BP_ND_ from baseline to intervention were 7.4 ± 16.0% after citalopram/pindolol and 0.6 ± 7.2% after ATD (Fig. 1). Unpaired *t*-tests revealed no statistically significant differences in percent change of neocortical [^11^C]Cimbi-36 BP_ND_ between placebo and citalopram/pindolol (*t*(9.7) = 0.7, *p* = 0.4), or between placebo and ATD (*t*(12.9) = 1.6, *p* = 0.5). Similar results were found when 2-TCM was used to estimate [^11^C]Cimbi-36 BP_ND_ (Supplementary Figure S2). As seen in Fig. 1, there was large within-subject variation of [^11^C]Cimbi-36 binding in the citalopram/pindolol group compared with placebo (Levene’s test F(1) = 9.9, *p* = 0.007). The change in neocortical BP_ND_ from baseline to intervention in each subject are shown in Supplementary Figures S3–S5.

Post hoc regional analyses revealed a significant difference in percent change of hippocampal [^11^C]Cimbi-36 BP_ND_ between placebo and Citalopram/Pindolol (t(13.7) = 2.4, *p* = 0.03, Supplementary Figure S6–S7), but not between placebo and ATD (t(5.8) = -0.7, *p* = 0.5). The mean percent difference in hippocampal BP_ND_ from baseline to intervention in the Citalopram/Pindolol group was −25.5 ± 25.2%, indicating increased serotonin levels in this region. We found no significant group differences in percent change of [^11^C]Cimbi-36 BP_ND_ in any of the cortical regions of interest (frontal, temporal and parietal cortex, anterior and posterior cingulate).

**Fig. 2:**
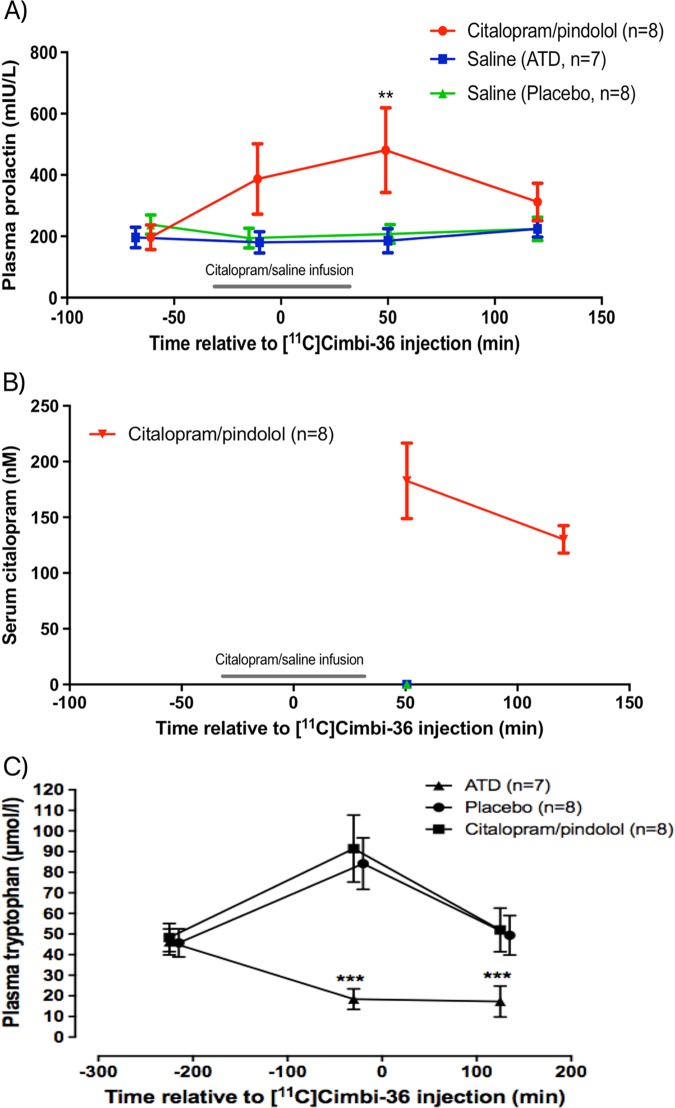
Effects of interventions on prolactin, citalopram and tryptophan levels. Time on the x-axis is referenced to the time of [^11^C]Cimbi-36 injection. **a** Plasma prolactin levels relative to time of [^11^C]Cimbi-36 injection. **p < 0.01 indicates Bonferroni-corrected post-test between groups (citalopram/pindolol versus ATD as well as placebo groups) in a 2-way ANOVA of prolactin concentration with time and treatment as main effects. Normal interval for plasma prolactin: 70-440mIU/L. **b** Serum citalopram concentrations relative to time of [^11^C]Cimbi-36 injection. Citalopram was given intravenously for one hour starting 30 minutes before time of injection. **c**. Plasma tryptophan levels relative to time of [^11^C]Cimbi-36 injection. ***p < 0.001 indicate Bonferroni-corrected post-test between groups (ATD versus citalopram/pindolol as well as placebo groups) in a 2-way ANOVA of tryptophan concentrations with time and treatment as main effects

The effects of citalopram/pindolol were confirmed by significant elevation of plasma prolactin measured one hour after SSRI infusion (*p* < 0.01, Fig. 2a), and serum citalopram measured 50 and 120min after radioligand injection (citalopram infusion started 30 min prior to radioligand injection and lasted for one hour) (Fig. 2b). Likewise, the effects of ATD were confirmed by significantly decreased concentrations of plasma tryptophan compared with subjects receiving the placebo drink containing all amino acids (*p* < 0.001, Fig. 2c). The mean percent change in the ratio of tryptophan to the other LNAAs (tryptophan load) from before to ~4 h after ingestion of the drink was −72.2 ± 7.2% in the ATD group, 40.4 ± 21.5% in the placebo group and 35.4 ± 7.2% in the citalopram/pindolol group.

**Fig. 3:**
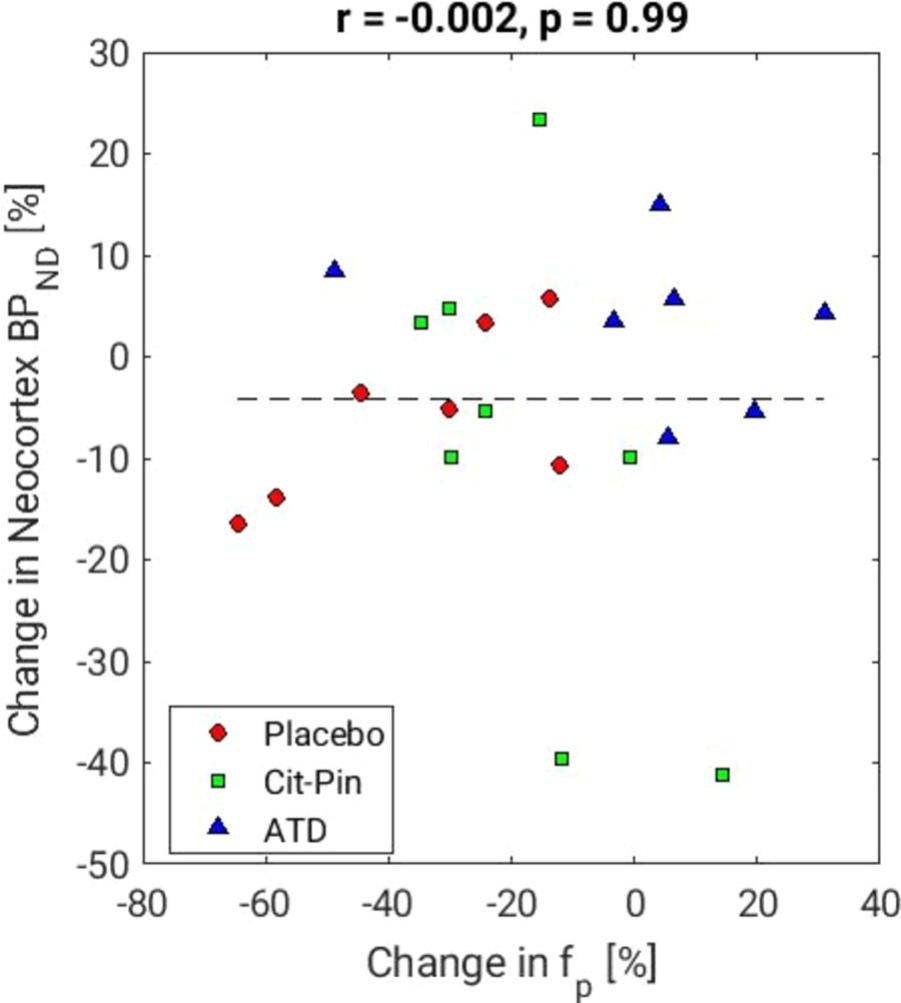
Association between the percent changes in *f*_P_ from baseline to intervention and percent change in neocortical BP_ND_ from baseline to intervention. Cit-Pin: citalopram/pindolol

Free radioligand fraction in plasma (*f*_P_) was significantly higher at intervention compared with baseline in the two groups receiving placebo amino acid drinks (Supplementary Figure S8). This change in *f*_P_ was not associated with changes in BP_ND_ from baseline to intervention across groups (slope estimate: −0.002, 95% confidence interval: [−0.28; 0.28], *p* = 0.99, Fig. 3), nor in each group analyzed separately (placebo group: *p* = 0.09, ATD group: *p* = 0.99, citalopram/pindolol group: *p* = 0.12).

## Discussion

This study examined the sensitivity of the 5-HT_2A_ receptor agonist radioligand [^11^C]Cimbi-36 to changes in endogenous serotonin concentrations in the human brain. Contrary to our hypothesis, neither ATD nor a combination of citalopram and pindolol elicited unidirectional changes in serotonin levels that were detectable with [^11^C]Cimbi-36 PET, at least not with the current sample size. Interestingly, we observed large within-group variation in [^11^C]Cimbi-36 binding after intervention with citalopram and pindolol as compared to placebo and ATD.

Studies in pigs and nonhuman primates show reductions in [^11^C]Cimbi-36 binding after interventions that increase serotonin levels^4,17^. Using microdialysis and [^11^C]Cimbi-36 PET in pigs, the potent serotonin releaser fenfluramine produced an increase in extracellular serotonin levels of 1123% relative to baseline, whereas citalopram in combination with pindolol led to an increase of 441% of serotonin baseline level, corresponding to a 5-HT_2A_ receptor occupancy of 44% with fenfluramine and 28% with citalopram and pindolol measured with [^11^C]Cimbi-36^17^. This correlation between pharmacologically induced changes in extracellular serotonin levels and changes in [^11^C]Cimbi-36 PET signal indicates that at least in pigs, [^11^C]Cimbi-36 is sensitive to changes in endogenous serotonin levels. In nonhuman primates, fenfluramine also decreased [^11^C]Cimbi-36 binding by 26–62%^4^. Given these observations in animals, we expected that [^11^C]Cimbi-36 binding would be reduced following intervention with citalopram/pindolol also in humans and we suspect that when given acutely, and in the doses used here, citalopram has variable effects on serotonin levels in the brain. Other human PET studies evaluating the effects of acute intervention with SSRI support this interpretation; two recent studies reported increased [^11^C]CUMI-101 (5-HT_1A_ receptor radioligand) and [^11^C]AZ10419369 (5-HT_1B_ receptor partial agonist) binding in projection areas after acute SSRI intervention (10 mg citalopram intravenously and a single oral dose of 20 mg escitalopram, respectively) suggesting a reduction of serotonin levels^6,7^. The authors suggested that the increase in cortical 5-HT_1A_ receptor binding after citalopram is driven by an action in the dorsal raphe nuclei, i.e., acute SSRI produces increases in serotonin levels in the raphe nuclei which then decreases firing rates in postsynaptic regions^6^. Microdialysis and neurophysiological studies in rodents have shown an increase in serotonin concentration in the raphe nuclei after administration of an SSRI, leading to attenuated release of serotonin in projection areas^18,19^ interpreted as caused by 5-HT_1A_ autoreceptor regulation. One speculation is that the autoregulation of serotonergic firing may be particularly strong in the human brain, which was also the reason for our decision to inhibit the raphe nuclei-mediated response to SSRI by blocking the 5-HT_1A_ autoreceptors with pindolol. However, the chosen dose of pindolol may only have been sufficient in some of the participants, which would be consistent with the larger within-subject variation of [^11^C]Cimbi-36 binding compared with placebo. Alternatively, we did manage to block the autoreceptors sufficiently, but there was a large interindividual variability in brain serotonin levels in response to SSRIs. In the latter case, our observation has a potential impact for the understanding of patient responses to SSRI. For example, SSRI treatment ameliorates depressive symptoms in some patients but not all^20,21^. The current sample size does not permit a meaningful analysis of potential causes of the larger variation in the citalopram/pindolol group, but may involve genetic variants, differences in pharmacodynamics, or other factors.

We used a global neocortical region in which [^11^C]Cimbi-36 binds selectively to 5-HT_2A_ receptors. In our post hoc regional analyses, we found a significant decrease in hippocampal BP_ND_ after Citalopram/Pindolol. This suggests that in hippocampus, Citalopram/Pindolol is associated with an increase in serotonin that is subject to less interindividual variation. Interestingly, hippocampal volume in depressed patients increased after treatment with citalopram^22^. Alternatively, the decline in hippocampal BP_ND_ could be caused by its high density of 5-HT_2C_ receptors. Recent studies have found that [^11^C]Cimbi-36 (in addition to 5-HT_2A_ receptors) also binds to 5-HT_2C_ receptors in hippocampus in the monkey^23^ and human brain^9^.

ATD is an established method for manipulating brain serotonin function, with effects on cognition and behavior^24^. In rodents, acute and chronic tryptophan depletion reduce central serotonin levels^25–28^. Using this technique, we compared [^11^C]Cimbi-36 BP_ND_ following ATD and a placebo condition, hypothesizing that if [^11^C]Cimbi-36 binding is susceptible to competition with endogenous serotonin, we would observe an increase in [^11^C]Cimbi-36 BP_ND_ after ATD. We found no significant changes in BP_ND_, suggesting that the reduction in endogenous serotonin levels elicited by ATD was not sufficient to produce a measurable increase in the binding of [^11^C]Cimbi-36. Indeed, the effect of ATD on central serotonin levels has been debated^29,30^, and the extent by which ATD alters serotonin levels in humans may vary across individuals and contexts^30^. Previous PET studies investigating the effects of ATD on components of the serotonin system have produced mixed results. Studies in healthy controls report no ATD induced changes in 5-HT_2A_ receptor binding ([^11^C]MDL100907)^31^, or serotonin transporter binding ([^11^C]DASB)^32,33^, whereas prefrontal monoamine oxidase A density was decreased following ATD^34^. In a study of patients with major depressive disorder, regional 5-HT_1A_ receptor binding ([^18^F]-MPPF) was not altered after ATD, although six of the eight patients had a transient relapse in depressive symptoms^35^. Studies examining patients with remitted major depressive disorder found that ATD induced a transient return of depressive symptoms^36,37^, along with decreased 5-HT_2_ receptor binding of [^18^F]Setoperone in patients^36^, and augmented regional cerebral glucose utilization in patients but not in controls^37^. We did not observe any effects of ATD on mood in our healthy participants (reported in Stenbæk et al.^10^). One interpretation of the current data is that these healthy participants, who showed no signs of psychopathology, may be resilient to ATD and are able to maintain stable serotonin levels and mood. Alternatively, ATD did not elicit a sufficient decrease in serotonin levels to be detected with our sample size.

On intervention days, all participants regardless of treatment group received a drink with a high load of amino acids. In the two groups receiving placebo drinks (containing tryptophan) radioligand plasma protein binding was lower on intervention days compared with baseline. Tryptophan in plasma is bound to albumin, with approximately 5% being left free and available for transport into the brain^13^. We suggest that the high load of amino acids, in particular tryptophan, was responsible for the increase in *f*_P_. A possible explanation is that competition between tryptophan and [^11^C]Cimbi-36 binding with plasma binding proteins (e.g. albumin) occurs, resulting in a larger proportion of free [^11^C]Cimbi-36 in plasma. Few other PET studies have reported effects of tryptophan on *f*_P_. Talbot et al.^31^ report that the *f*_P_ of [^11^C]MDL100907 did not differ between placebo and ATD^31^. In another study investigating binding of the serotonin transporter tracer [^11^C]DASB after intervention with ATD, there was no difference in *f*_P_ of [^11^C]DASB between the two conditions, but there was a small decrease in both V_T_ and BP from baseline to intervention that was related to a concomitant difference in *f*_P_ of similar magnitude^32^. In the latter study, the change in BP and V_T_ became non-significant when corrected for *f*_P_, suggesting that the effect may have been driven by changes in *f*_P_^32^. We used BP_ND_ as the outcome measure; this parameter is independent of changes in *f*_P_, and as such, we consider the findings of this study to reflect changes in 5-HT_2A_ receptor availability.

A few limitations of this study should be mentioned. The study includes a limited sample size, but the low test-retest variability and investigation of the same individuals before and after the intervention is a strong design. Even though we cannot exclude that the variability observed in the citalopram/pindolol group can be attributed to measurement error we find this unlikely, given the low variability in the placebo group. Another limitation is that the increase in both the tryptophan concentration and the tryptophan load (Trp/ΣLNAA ratio) in the groups receiving placebo amino acid drinks may have affected the cerebral serotonin levels in these participants, potentially interfering with the effects of SSRI treatment. Likewise, pindolol treatment may also have affected the cerebral serotonin levels, putatively interfering the effect of SSRI.

In conclusion, the data presented in this study suggest that neither ATD nor a combination of citalopram and pindolol elicit acute unidirectional and global changes in extracellular serotonin levels that are sufficient to be detected by [^11^C]Cimbi-36 PET. Recent data suggest that [^11^C]Cimbi-36 is sensitive to changes in endogenous serotonin levels only when serotonin release is sufficiently high, e.g. amphetamine challenge^38^. The large variability in [^11^C]Cimbi-36 binding after citalopram/pindolol could reflect interindividual differences in brain serotonin levels after an acute SSRI intervention. This observation may have a potential impact for the understanding of patient responses to SSRIs, which should be explored in future studies.

## Supplementary information

Supplementary Information
