# Supplementary material for: Measuring endogenous changes in serotonergic neurotransmission with [11C]Cimbi-36 positron emission tomography in humans
Source: Transl Psychiatry. 2019 Apr 11;9:134. doi: 10.1038/s41398-019-0468-8 (PMC6459901; doi:10.1038/s41398-019-0468-8)
Supplement: Supplementary file 1 — Supplementary Information [file 41398_2019_468_MOESM1_ESM.docx]

**Supplementary Information**

**Measuring endogenous changes in serotonergic neurotransmission with [^11^C]Cimbi-36 PET in humans**

***Authors:*** *Sofi da Cunha-Bang, Anders Ettrup, Brenda Mc Mahon, Anine Persson Skibsted, Szabolcs Lehel, Agnete Dyssegaard, Louise Møller Jørgensen, Kirsten Møller, Nic Gillings, Martin Schain, Claus Svarer and Gitte M. Knudsen*

**Supplementary Methods**


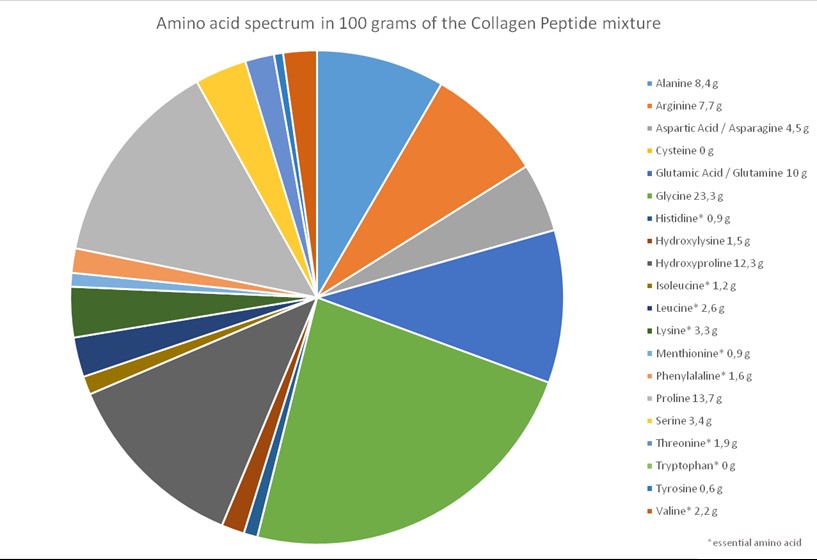


**Figure S1**. Amino acid content of the Solugel 5000® mixture.

***Diet during intervention days***

10.00 a.m.: 2 fruits

11.40 a.m.: 2 crispbreads, 15 g butter, 25 gr brie cheese, 20 g marmalade, 1 fruit

3.00 p.m.: 1 crispbread, 15 g butter, 1 fruit.

Participants were given maximum one banana/day.

***Written instructions to participants of which foods to avoid from 3.00 p.m. on the day before intervention scanning***

Not allowed to eat: meat, fish, eggs, beans, nuts, seeds, tofu and soya products, chocolate and cacao, oatmeal.

Allowed to eat in limited amounts (maximum two of the following products): 2 slices of cheese, 25 g brie cheese (or corresponding cheese), 50 g cottage cheese, 50 g goats cheese, 1 glass of milk, 50 ml cream, 1 glass/small bowl of yoghurt.

Allowed to eat: fruits, vegetables, vegetable/tomato soup, rice, crispbreads, ryebread with low amounts of seeds, marmalade, butter/oil.

***Analysis of amino acids in blood***

Blood was drawn from an elbow vein into a heparinized vial and stored on ice until centrifugation (10 min. at 3000 rpm, 4 ˚C). A fraction of the plasma phase (0.5 ml) was mixed with an equal volume of sulfosalicylic acid containing norleucine as internal standard to precipitate plasma proteins. After a second centrifugation (30 min. at 3000 rpm, 4 ˚C), the supernatant fluid was collected and stored at -80 ˚C until analyzed. High performance liquid chromatography was used to measure tryptophan (Trp) concentrations in the blood samples. Concentrations of other large neutral amino acids (LNAAs) were also measured to obtain a Trp/LNAA ratio as Trp competes with those for the same transporter system to enter the brain.

**Supplementary Results**

**Figure S2.** Effects of citalopram/pindolol and acute tryptophan depletion (ATD) intervention on neocortical [^11^C]Cimbi-36 binding potential (BP_ND_) calculated using 2 tissue compartment modeling (2-TCM). Difference in outcome is calculated as (BP_ND_^intervention^ - BP_ND_^baseline^)/ BP_ND_^baseline^ for each subject.

**Figure S3.** Neocortical 5-HT_2A_ receptor binding (BP_ND_) at baseline and intervention in eight subjects who received Citalopram/Pindolol.

**
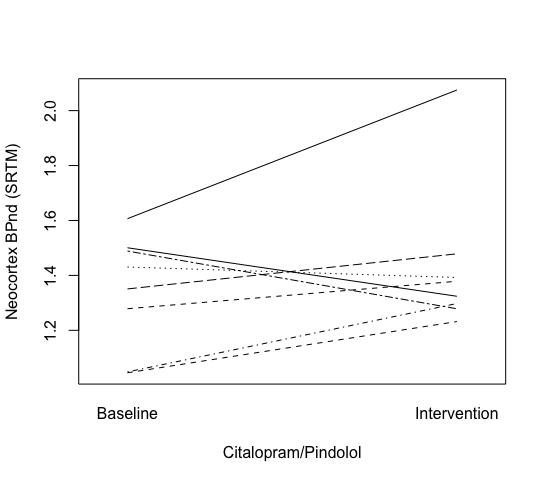
**

**Figure S4.** Neocortical 5-HT_2A_ receptor binding (BP_ND_) at baseline and intervention in seven subjects who received acute tryptophan depletion (ATD).

**
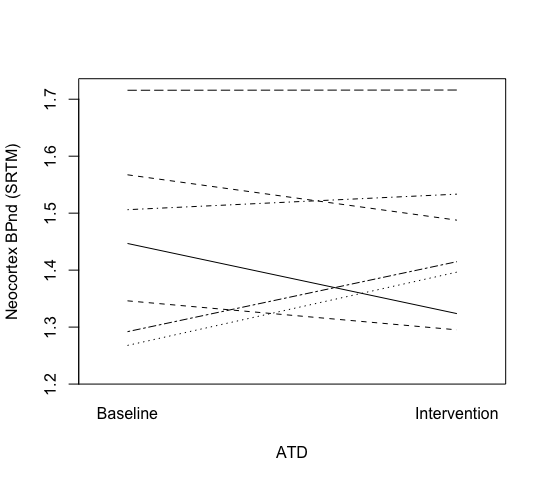
**

**Figure S5.** Neocortical 5-HT_2A_ receptor binding (BP_ND_) at baseline and intervention in eight subjects who received placebo.

**
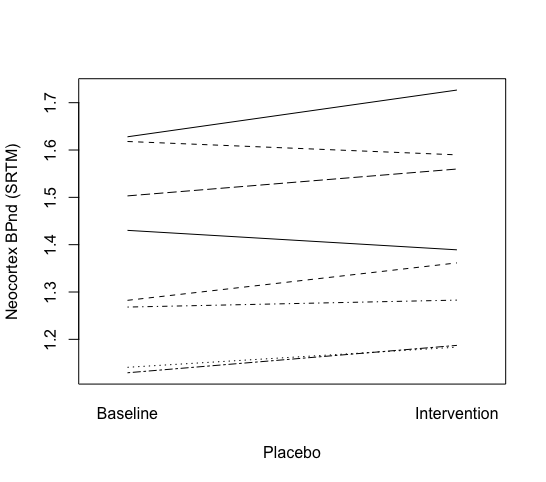
**

**Figure S6.** Hippocampal 5-HT_2A_ receptor binding (BP_ND_) at baseline and intervention in eight subjects who received Citalopram/Pindolol.

**
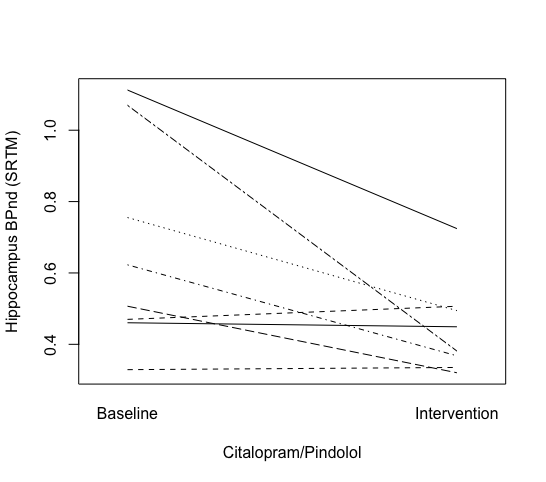
**

**Figure S7.** Effects of citalopram/pindolol (SSRI) and acute tryptophan depletion (TD) compared to placebo (PLC) on hippocampus [^11^C]Cimbi-36 binding potential (BP_ND_). Difference in outcome is calculated as (BP_ND_^intervention^ - BP_ND_^baseline^)/ BP_ND_^baseline^ for each subject. Compared to placebo, there was a significant decrease in [^11^C]Cimbi-36 BP_ND_ after Citalopram/Pindolol (t(13.7)=2.4, p=0.03), but not after ATD (t(7.3)=-0.5, p=0.6).

**
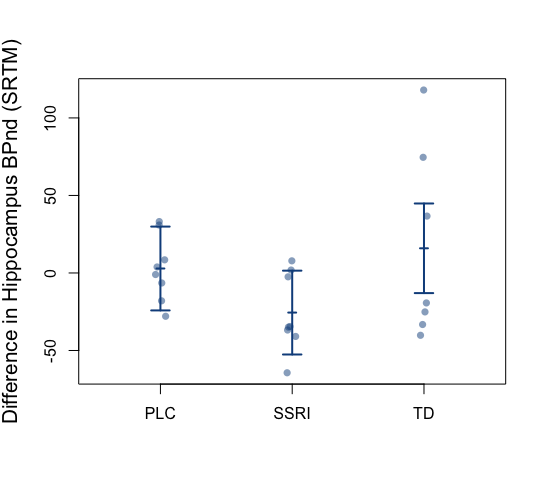
**

**Figure S8.** Free fraction of radioligand in plasma (*f*_P_) at baseline and intervention scans. Titles show average (SD) percent difference between intervention and baseline, and p-values were calculated using paired t-tests.

**
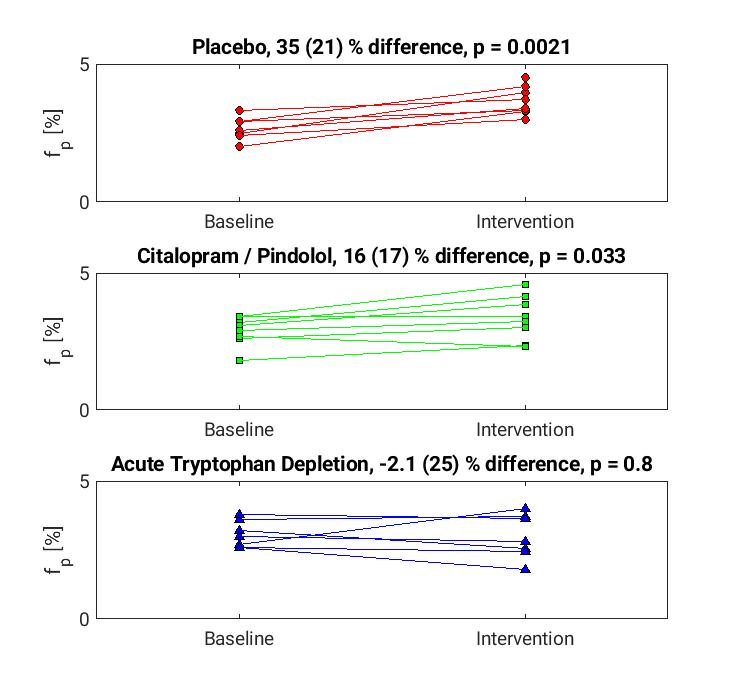
**
